# Antibody-blocking of a tick transporter impairs* Anaplasma phagocytophilum* colonization in *Haemaphysalis longicornis* ticks

**DOI:** 10.1038/s41598-024-59315-w

**Published:** 2024-04-18

**Authors:** Prachi Namjoshi, Donald M. Lubembe, Hameeda Sultana, Girish Neelakanta

**Affiliations:** 1 Department of Biomedical and Diagnostic Sciences, College of Veterinary Medicine, University of Tennessee, Knoxville, TN 37996 USA; 2 Department of Veterinary Pathology, Microbiology and Parasitology, Faculty of Veterinary Medicine and Surgery, Egerton University, Egerton, Kenya

**Keywords:** *Haemaphysalis longicornis*, *Ixodes scapularis*, Organic anion transporting polypeptide (OATP), Tryptophan pathway, Xanthurenic acid (XA), Antibody-blocking, Vaccine, Microbiology, Diseases

## Abstract

The invasive Asian longhorned tick *Haemaphysalis longicornis* that vectors and transmits several animal pathogens is significantly expanding in the United States. Recent studies report that these ticks also harbor human pathogens including *Borrelia burgdorferi* sensu lato, *Babesia microti*, and *Anaplasma phagocytophilum*. Therefore, studies that address the interactions of these ticks with human pathogens are important. In this study, we report the characterization of *H. longicornis* organic anion-transporting polypeptides (OATPs) in interactions of these ticks with *A. phagocytophilum*. Using OATP-signature sequence, we identified six OATPs in the *H. longicornis* genome. Bioinformatic analysis revealed that *H. longicornis* OATPs are closer to other tick orthologs rather than to mammalian counterparts. Quantitative real-time PCR analysis revealed that OATPs are highly expressed in immature stages when compared to mature stages of these ticks. In addition, we noted that the presence of *A. phagocytophilum* upregulates a specific OATP in these ticks. We also noted that exogenous treatment of *H. longicornis* with xanthurenic acid, a tryptophan metabolite, influenced OATP expression in these ticks. Immunoblotting analysis revealed that antibody generated against *Ixodes scapularis* OATP cross-reacted with *H. longicornis* OATP. Furthermore, treatment of *H. longicornis* with OATP antibody impaired colonization of *A. phagocytophilum* in these ticks. These results not only provide evidence that the OATP-tryptophan pathway is important for *A. phagocytophilum* survival in *H. longicornis* ticks but also indicate OATP as a promising candidate for the development of a universal anti-tick vaccine to target this bacterium and perhaps other rickettsial pathogens of medical importance.

## Introduction

*Haemaphysalis longicornis,* also known as the Asian longhorned tick, is a hard tick and native in eastern Asia, Australia, New Zealand, and several pacific islands^1,2^. The first recognized human bite of *H. longicornis* was reported in 2019^3^. This tick is reported to vector at least 30 human pathogens^4^. In Asia, *H. longicornis* ticks have been reported to serve as vector for various pathogens like spotted fever group rickettsia, *Anaplasma* species, *Borrelia burgdorferi* sensu lato, and *Babesia* species^5,6^. This tick was recently found in the United States^7^. The detection of human pathogenic variant of *A. phagocytophilum* was first reported in *H. longicornis* that were field collected in Pennsylvania, USA^8^. A recent study reported that 8% of field collected *H. longicornis* ticks from Ohio, USA, were positive for *A. phagocytophilum*^9^. In addition, another recent study reported the presence of *B. burgdorferi* sensu lato and *B. microti* in field collected ticks from Pennsylvania, USA^10^. More recent reports show that *Ehrlichia chaffeensis*, *Anaplasma bovis* and spotted fever group rickettsiae were also detected in *H. longicornis* ticks^11,12^. Recently, human pathogenic *A. phagocytophilum* variant was detected in field collected *H. longicornis* ticks in USA^8,9^. *Haemaphysalis longicornis* ticks have four stages of development (egg, larvae, nymph, adult). Larvae, nymphs and adults require a blood meal to molt. These ticks could feed on a wide variety of hosts including rodents, livestock, carnivores, and birds^1^. *Haemaphysalis longicornis* ticks can reproduce bisexually or asexually by parthenogenesis^1,2,13^ making them highly invasive species. Modeling study has predicted that eastern North America from southern Canada to the Gulf Coast, small temperate area on the West Coast, Midwestern and Southern United States habitats are suitable for *H. longicornis*^14^. Due to the ability of this tick to transmit multiple pathogens to wide range of vertebrate hosts including pets and livestock, the invasion and dominance of *H. longicornis* infestations are of medical and veterinary concern.

*Anaplasma phagocytophilum*, an obligate intracellular bacterium, is a causative agent of human granulocytic anaplasmosis (HGA)^15,16^. In the United States, *I. scapularis*, also known as a deer tick, and *Ixodes pacificus* are known vectors to transmit *A. phagocytophilum* to the vertebrate hosts^17–20^. This bacterium infects diverse hosts and is reported to modulate various signaling cascades in ticks and in vertebrates hosts^17,21–28^. Organic anion transporting polypeptides (OATPs) are transmembrane proteins that aid in the movement of various anions, hormones, drugs, signaling molecules, growth factors and toxins^29–33^. Our previous studies reported that *A. phagocytophilum* modulates *I. scapularis* OATPs and tryptophan pathway not only for its survival but also for its transmission from these ticks to vertebrate host^34–40^. *Anaplasma phagocytophilum* upregulates endogenous production of xanthurenic acid (XA), a tryptophan metabolite^37^, increases kynurenine amino transferase (KAT) activity^34,40^, upregulates expression of IsOATP4056 transcripts and protein^36,40^, modulates ROS production^34^ and activates p38 MAPK pathway for its survival in the vector host^37^. We have previously noted that exogenously added XA can induce *isoatp4056* expression and increases the bacterial burden in *A. phagocytophilum* infected *I. scapularis* ticks and tick cells^40^. In addition, we reported that OATPs are important for survival of tick-borne viruses in *I. scapularis* ticks^41^. Overall, these studies indicate that OATPs are important for intracellular pathogen survival in *I. scapularis* ticks.

OATPs have several transmembrane domains, intracellular and extracellular loops^36,41^. In our previous study, we generated affinity purified antibodies against the C-terminal extra-cellular loop 6 (EL-6) of *I. scapularis* IsOATP4056 protein^36^. We reported that when mice were passively immunized with an anti-EL6 antibody there was a significant decrease in the transmission of *A. phagocytophilum* from infected ticks to the murine host^36^. In addition, we reported that ticks ingested EL-6 antibody via a blood meal. Anti-EL6 antibodies in a blood meal affected *A. phagocytophilum* loads in fed ticks^36^. We also noted that treatment of tick cells with an anti-EL6 antibody affected *A. phagocytophilum* growth in these cells^36^. Collectively, these results not only indicate that anti-EL6 antibodies could be effective in impairing transmission of *A. phagocytophilum* from infected ticks to the naïve vertebrate host but also aid in clearing bacterial loads in ticks.

In this study, we not only provide evidence that *H. longicornis* ticks expresses OATPs, but also report that these OATPs are modulated by *A. phagocytophilum* in these ticks. We also report a method for the generation of *A. phagocytophilum*-infected *H. longicornis* ticks in vitro. We noted that treatment of *H. longicornis* unfed nymphs with an anti-EL6 antibody decreased bacterial burden in these ticks. This study provide evidence that EL6 region of OATP could be envisioned as an important candidate for the development of universal anti-vector vaccine to target *A. phagocytophilum* and perhaps other intracellular pathogens in hard ticks.

## Results

### Expression of *H. longicornis* organic anion transporting polypeptides (OATPs) transcripts

**Figure 1:**
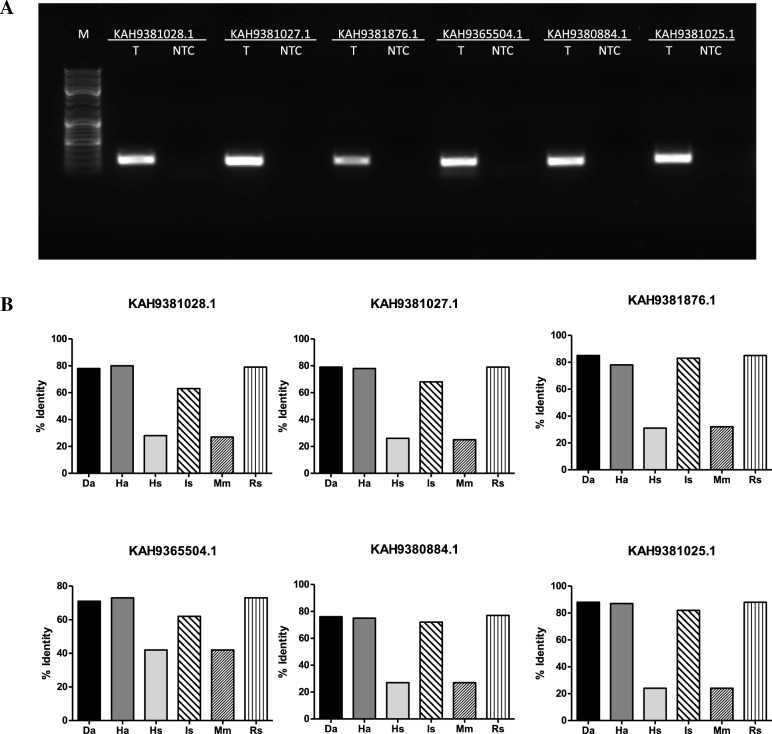
PCR amplification and sequence analysis of *H. longicornis oatp* genes. (**A**) PCR amplification products of *H. longicornis oatps* is shown. M indicates the DNA marker and NTC indicates no template control. T indicates cDNA template generated from unfed *H. longicornis* nymphal tick RNA. GenBank accession numbers are labeled at the top of the gel image. (**B**) Bar graphs (with different color shades) represents percent identity of *H. longicornis* OATPs amino acid sequence with ortholog proteins from *Dermacentor andersoni* (*Da*), *Hyalomma asiaticum* (*Ha*), *Homo sapiens* (*Hs*)*, I. scapularis* (*Is*), *Mus musculus* (*Mm*) and *Rhipicephalus sanguineus* (*Rs*). GenBank accession numbers for all OATPs are shown in Supplementary Table 3.

OATPs have a signature motif (WxGxWWxG) in the amino acid sequence^41^. We first screened the genome of *H. longicornis* and identified six OATPs (Supplementary Table 1). To analyze whether *H. longicornis* ticks express these OATPs, RNA from unfed uninfected adult ticks was used as a template for reverse transcription polymerase chain reaction (RT-PCR). Using oligonucleotides mentioned in Supplementary Table 2, we amplified all six OATP transcripts (Fig. 1A). The RT-PCR products were excised and sequenced to confirm the nucleotide sequence of all six OATPs. Amino acid percent identity analysis of *H. longicornis* OATP sequence with other ortholog proteins from *Dermacentor andersoni* (*Da*), *Hyalomma asiaticum* (*Ha*), *Homo sapiens* (*Hs*)*, I. scapularis* (*Is*), *Mus musculus* (*Mm*) and *Rhipicephalus sanguineus* (*Rs*) was determined by CLUSTALW alignment (Fig. 1B). The GenBank accession numbers for these proteins are shown in Supplementary Table 3. The *H. longicornis* OATPs (KAH9381028.1, KAH9381027.1, KAH9381876.1, KAH9365504.1, KAH9380884.1, KAH9381025.1) share 62–88% identity with OATPs from other ticks analyzed in this study but 24–42% identity with OATPs from human and mice (Fig. 1B, Supplementary Figs. 1 and 2). Furthermore, phylogenetic analyses revealed that *H. longicornis* OATP amino acid sequences fall in the same clade or clade close to OATPs from other ticks (Supplementary Figs. 3 and 4). Both human and mouse OATPs form a different clade (Supplementary Figs. 3 and 4). These results reveal that *H. longicornis* OATPs are similar to OATPs from other ticks.

### Comparison of OATP-signature sequence and prediction of posttranslational modifications on *H. longicornis* OATPs

**Figure 2:**
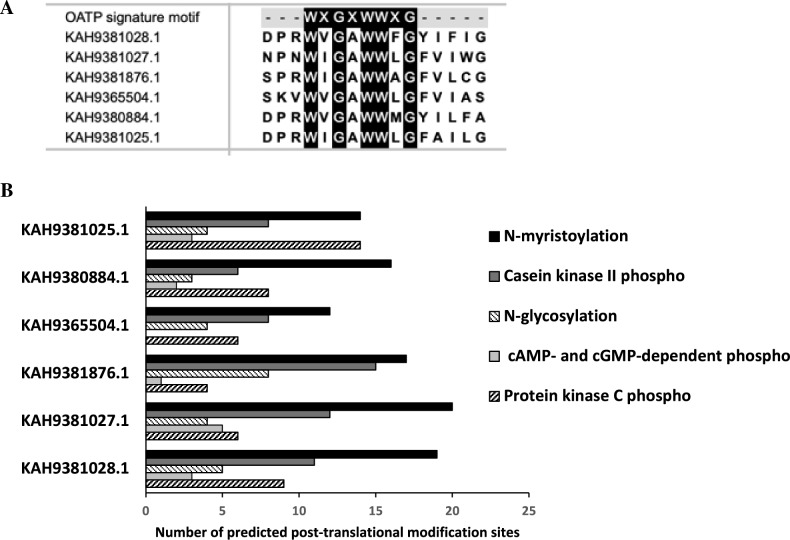
Alignment of *H. longicornis* OATPs with the OATP-signature sequence and analysis of post-translational modifications on *H. longicornis* OATPs. (**A**) CLUSTALW alignment of consensus OATP-signature sequence (WxGxWWxG) with all *H. longicornis* OATPs. Alignment was performed with DNASTAR MEGALIGN software. Residues that match are shaded in black. (**B**) Number of predicted post-translational sites (shown on X-axis) in *H. longicornis* OATPs were analyzed at PROSITE. Histograms represent the number of N- myristoylation, casein kinase II phosphorylation, N-glycosylation, cAMP or c-GMP-dependent protein kinase phosphorylation and protein kinase C phosphorylation sites for each OATP. Full-length *H. longicornis* OATP sequences were considered to determine posttranslational modification sites.

The alignment of the amino acid sequences of *H. longicornis* OATPs with the OATP signature sequence WxGxWWxG^41^ using ClustalW program revealed a conserved OATP signature motif in all six *H. longicornis* OATPs (Fig. 2A). Bioinformatic analysis of post translational modification sites in *H. longicornis* OATPs revealed that KAH9381027.1 had higher number of N-myristoylation sites (20 sites) and cAMP- and cGMP-dependent phospho sites (five sites) compared to other OATPs (Fig. 2B). KAH9381876.1 OATP had higher number of casein kinase II phospho sites (15 sites) and N-glycosylation (eight sites). KAH9381025.1 had the higher number of protein kinase C phospho sites (14 sites) compared to other OATPs. All but XP_040071371 of *I. scapularis* OATP orthologs had at least one of each of these posttranslational modification sites (Supplementary Table 4). Collectively, these analyses show that like I. scapularis, *H. longicornis* OATPs also have several posttranslational modification sites in their amino acid sequences.

### Expression of *H. longicornis* OATPs is developmentally regulated

**Figure 3:**
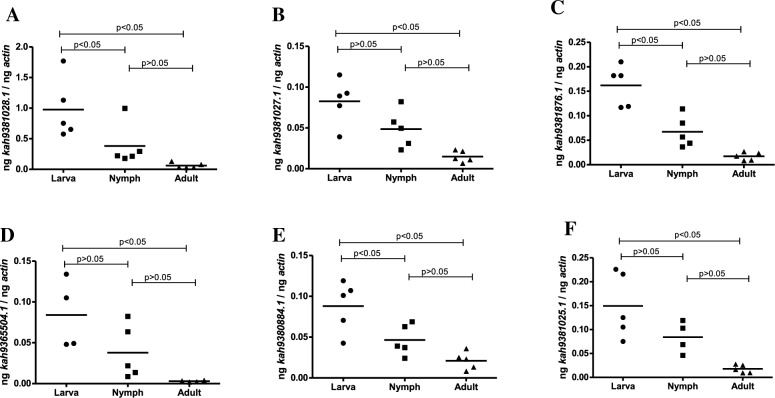
Expression of *H. longicornis oatp* transcripts at different tick developmental stages*.* Quantitative RT-PCR analysis showing expression of *kah9381028.1* (**A**), *kah9381027.1* (**B**), *kah9381876.1* (**C**), *kah9365504.1* (**D**), *kah9380884.1* (**E**), *kah9381025.1* (**F**) at different tick developmental stages in uninfected unfed *H. longicornis*. Each data point in nymphs and adult samples represents transcript levels noted in an individual tick. Closed circles, squares and triangles represent larvae, nymphs and adults, respectively. For larval samples, each data point represents transcript levels noted in five pooled larvae. Horizontal lines in the graphs represent the mean value of the data points. The mRNA levels of these genes were normalized to tick beta-actin mRNA levels. Statistical significance was calculated using ANOVA analysis.

We then analyzed whether all six OATP transcripts are expressed in different tick developmental stages (Fig. 3). qRT-PCR analysis showed that larvae expressed significantly (P < 0.05) higher levels of *kah9381028.1* (Fig. 3A), *kah9381027.1* (Fig. 3B), *kah9381876.1* (Fig. 3C), *kah9365504.1* (Fig. 3D), *kah9380884.1* (Fig. 3E), *kah9381025.1* (Fig. 3F) in comparison to levels noted in adult ticks. Larvae expressed significantly (P < 0.05) higher levels of *kah9381028.1*, *kah9381876.1* and *kah9380884.1* mRNA in comparison to nymphs (Fig. 3A, C, E). No significant (P > 0.05) differences in *H. longicorinis oatp* mRNA levels were observed between nymph and adults (Fig. 3A–F). However, we observed a decreased trend in the expression of OATPs in adults compared to nymphs (Fig. 3A–F). These data show variable expression pattern of OATP transcript levels at different developmental stages of *H. longicornis* ticks.

### Generation of *A. phagocytophilum*-infected *H. longicornis* ticks in vitro

**Figure 4:**
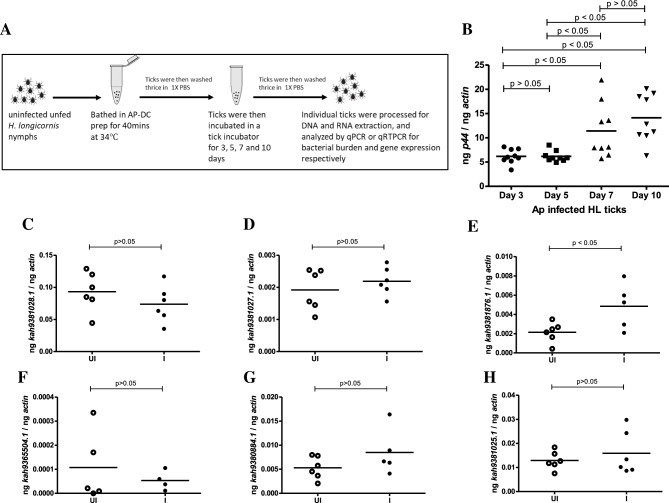
*Anaplasma phagocytophilum* up-regulates expression of *kah9381876.1* mRNA levels in unfed *H. longicornis* nymphs. (**A**) Schematic representation showing in vitro infection of *H. longicornis* nymphal ticks with *A. phagocytophilum*. (**B**) qPCR with tick DNA showing levels of *A. phagocytophilum* in infected *H. longicornis* ticks at different days post-infection. Statistical significance was calculated using ANOVA analysis. qRT-PCR analysis showing expression of kah*9381028.1* (**C**), *kah9381027.1* (**D**), *kah9381876.1* (**E**), *kah9365504.1* (**F**), *kah9380884.1* (**G**), *kah9381025.1* (**H**) in unfed uninfected or *A. phagocytophilum*-infected *H. longicornis* ticks. Open circles represent uninfected (UI) and closed circles represent infected (I) ticks. Each circle represents data from samples generated from one tick. Horizontal lines in the graphs represent the mean value of the data points. The mRNA levels of these genes were normalized to tick beta-actin mRNA levels. P value from student’s *t*-test is shown.

Pathogens like *Ehrlichia chaffeensis*, *A. bovis*^11^, Lyme spirochetes and spotted fever group rickettsiae^12^ have been detected in *H. longicornis* ticks. Recently, human pathogenic *A. phagocytophilum* variant was detected in field collected *H. longicornis* ticks in USA^8,9^. We reasoned whether expression of OATPs in *H. longicornis* is altered upon *A. phagocytophilum* infection. *Anaplasma phagocytophilum*-infected *H. longicornis* ticks were generated in vitro as shown in the schematic diagram (Fig. 4A). Briefly, dense core (DC) form of *A. phagocytophilum* was isolated from infected HL-60 cultures as described^42^. Uninfected unfed *H. longicornis* nymphal ticks were bathed in *A. phagocytophilum* DC culture for 40 min (Fig. 4A). Ticks were washed and incubated for 3, 5, 7 and 10 days and processed for DNA extraction. qPCR was performed with the DNA isolated from these bathed and washed ticks to detect *A. phagocytophilum* loads. The qPCR analysis revealed no significant (P > 0.05) differences in the bacterial loads between day 3 and day 5 post-infected ticks (Fig. 4B). However, significantly (P < 0.05) higher bacterial loads were evident in both day 7 and day 10 post-infected ticks in comparison to day 3 and day 5 post-infected ticks (Fig. 4B). No significant (P > 0.05) differences in the bacterial loads were evident between day 7 and day 10 post-infected (p.i.) ticks (Fig. 4B). The results not only indicate day 7 as a peak of infection time point in the in vitro *A. phagocytophilum* infection of *H. longicornis* ticks but also suggests that *A. phagocytophilum* multiply in *H. longicornis* ticks.

We then used *I. scapularis* ticks which is a known vector for this pathogen as a control group and performed in vitro infection like the one performed with *H. longicornis* ticks. The in vitro infected *I. scapularis* was incubated for 7 days post infection. qPCR analysis followed by agarose gel electrophoresis confirmed that like *H. longicornis*, *I. scapularis* ticks could also be infected with *A. phagocytophilum* by bathing these ticks in isolated DC cultures (Supplementary Fig. 5A, B). However, qPCR analysis revealed no significant difference in the *A. phagocytophilum* loads between *I. scapularis* and *H. longicornis* ticks at day 7 p.i. (Supplementary Fig. 5B).

### Anaplasma phagocytophilum induces expression of specific *H. longicornis* OATPs in unfed ticks

The impact of tick-borne pathogens on the expression of *H. longicornis* OATPs is not studied. Therefore, expression of OATPs was determined in the RNA samples generated from unfed *H. longicornis* nymphal ticks infected with *A. phagocytophilum* and compared the levels in uninfected ticks (Fig. 4C–H). qRT-PCR analysis revealed no significant differences in the expression levels of *kah9381028.1* (Fig. 4C), *kah9381027.1* (Fig. 4D), *kah9365504.1* (Fig. 4F), *kah9380884.1* (Fig. 4G) and *kah9381025.1* (Fig. 4H) between unfed uninfected ticks and *A. phagocytophilum*-infected ticks. However, expression of *kah9381876.1* (Fig. 4E) was significantly (P < 0.05) upregulated in the presence of *A. phagocytophilum* in comparison to the uninfected controls. These results show that *A. phagocytophilum* induces expression of specific tick OATPs (*kah9381876.1*) in unfed *H. longicornis* ticks.

### Xanthurenic acid (XA), a tryptophan metabolite, induces some of the OATPs expression in *A. phagocytophilum-*infected *H. longicornis* unfed nymphal ticks

**Figure 5:**
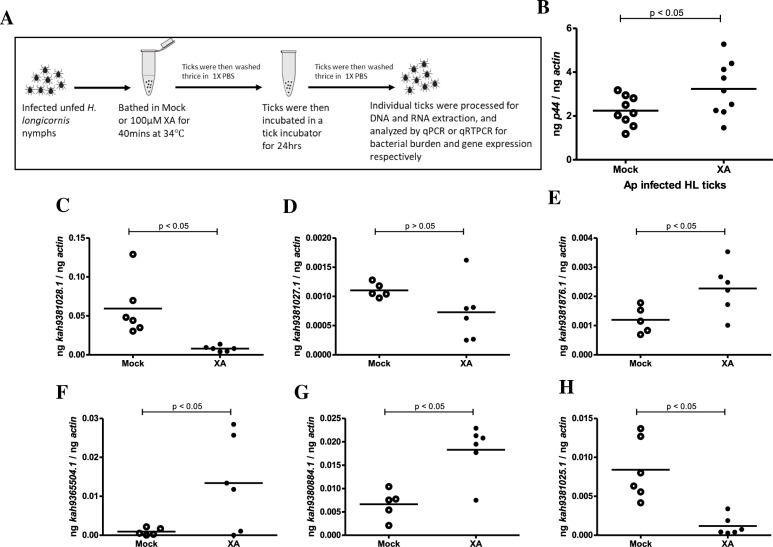
Exogenous treatment with XA differentially regulates some of the *oatps* in *A. phagocytophilum*-infected unfed *H. longicornis* nymphs. (**A**) Schematic representation showing the procedure for exogenous XA treatment (100 μM) of *A. phagocytophilum* infected *H. longicornis* nymphs. (**B**) qPCR with tick DNA showing bacterial burden in mock or XA-treated *A. phagocytophilum*-infected *H. longicornis* ticks. qRT-PCR analysis showing expression of *kah9381028.1* (**C**), *kah9381027.1* (**D**), *kah9381876.1* (**E**), *kah9365504.1* (**F**), *kah9380884.1* (**G**), *kah9381025.1* (**H**) in unfed *A. phagocytophilum*-infected *H. longicornis* ticks upon treatment with XA. Mock controls were treated with the same amount of solvent used for the preparation of XA. Open circles represent mock (M) and closed circles represent xanthurenic acid (XA)-treated *A. phagocytophilum* infected ticks. Each circle represents data from samples generated from one tick. Horizontal lines in the graphs represent the mean value of the data points. The mRNA levels of these genes were normalized to tick beta-actin mRNA levels. P value from student’s *t*-test is shown.

In our previous study, we noted higher levels of XA in *A. phagocytophilum*-infected *I. scapularis* ticks^37^. Therefore, we studied whether XA has any impact on bacterial burden and OATP expression in *A. phagocytophilum*-infected *H. longicornis* ticks. First, we generated *A. phagocytophilum*-infected *H. longicornis* ticks (day 7 post-infected) as described in Fig. 4A. These *A. phagocytophilum*-infected *H. longicornis* nymphs were bathed in 100 µM XA or mock solutions as depicted in the schematic diagram (Fig. 5A). After 24 h, post-bathed ticks were washed and processed for DNA or RNA extractions. qPCR with DNA samples revealed significantly (P < 0.05) increased bacterial burden in XA-treated *A. phagocytophilum*-infected *H. longicornis* ticks compared to the levels noted in mock-treated control ticks (Fig. 5B). qRT-PCR analysis with RNA samples revealed no significant differences in the expression levels of *kah9381027.1* (Fig. 5D) between mock- and XA-treated *A. phagocytophilum*-infected ticks. However, expression of *kah9381876.1* (Fig. 5E), *kah9365504.1* (Fig. 5F) and *kah9380884.1* (Fig. 5G) were significantly (P < 0.05) upregulated in XA-treated *A. phagocytophilum-infected H. longicornis* ticks in comparison to the mock-treated controls. Conversely, expression of *kah9381028.1* (Fig. 5C) and *kah9381025.1* (Fig. 5H) were significantly (P < 0.05) down-regulated in XA-treated *A. phagocytophilum-infected H. longicornis* ticks in comparison to the respective mock-treated controls. These results show that XA modulates expression of OATPs in *A. phagocytophilum-*infected *H. longicornis* ticks.

### Treatment with EL-6 antibody affects *A. phagocytophilum* burden in *H. longicornis* ticks

**Figure 6:**
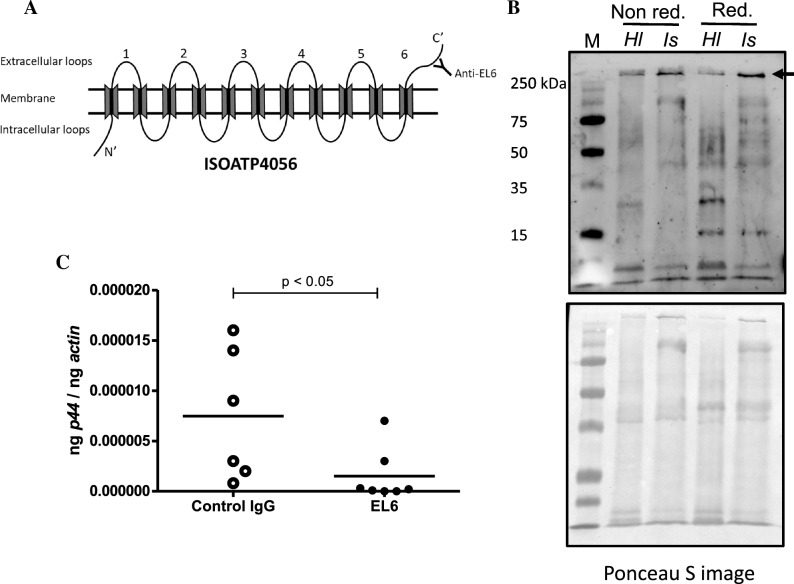
EL-6 antibody-treatment reduces bacterial burden in *A. phagocytophilum*-infected unfed *H. longicornis* nymphs. (**A**) Schematic representation of *I. scapularis* OATP organization on the tick cell plasma membrane is shown. Extracellular loops are indicated with numbers from 1 to 6. The N- and C-terminal ends are labeled. Antibody generated against epitope from *I. scapularis* EL-6 region was used in this study. Schematics are not drawn to the scale. (**B**) Immunoblotting analysis with EL-6 antibody showing levels of KAH9381876.1 or IsOATP4056 (indicated by black arrow) in uninfected unfed *H. longicornis* and *I. scapularis* tick lysates, respectively is shown. M indicates protein marker. Non red: indicates non-reducing conditions. Red: indicates reducing conditions, *Hl* indicates *H. longicornis*, and *Is* indicates *I. scapularis*. Ponceau S-stained gel image for total protein profile serves as loading control in the immunoblotting analysis. (**C**) qRT-PCR analysis showing bacterial burden (analyzed for *A. phagocytophilum p44* gene DNA levels) in control IgG or EL-6 antibody-treated *A. phagocytophilum*-infected unfed *H. longicornis* ticks. Control IgG and EL-6 antibodies were used at a concentration of 5 µg/ml. Open circles represent samples generated from control IgG-treated and closed circles represent EL-6 -treated *A. phagocytophilum*-infected unfed *H. longicornis* ticks. Each circle represents data from samples generated from one tick. Horizontal lines in the graphs represent the mean value of the data points. *A. phagocytophilum p44* levels were normalized to tick actin levels. P value from student’s *t*-test is shown.

Affinity purified polyclonal antibody was generated against IsOATP4056 Extracellular Loop-6 (EL-6) region^36^ (Fig. 6A). We reported that passive immunization with EL-6 antibody impaired *A. phagocytophilum* transmission from infected ticks to the naïve murine hosts^36^. In addition, treatment with EL-6 antibody affected *A. phagocytophilum* burden in tick cells^36^. *H. longicornis* OATP (*kah9381876.1*) is an ortholog of IsOATP4056. In this study, we noted that *kah9381876.1*, was significantly upregulated upon *A. phagocytophilum* infection in *H. longicornis* ticks (Fig. 4E). The epitope for EL-6 antibody binding in *I. scapularis* IsOATP4056^36^ is highly conserved in *H. longicornis* KAH9381876.1. Therefore, we used an anti-EL-6 antibody and performed immunoblotting and antibody-blocking experiments. Immunoblotting analyses with EL-6 antibody and 12% (Fig. 6B) or 8% (Supplementary Fig. 6) SDS-PAGE performed in non-reducing (no boiling) and reducing conditions revealed an intense band above 250 kDa in *H. longicornis* (Hl) whole tick lysates (Fig. 6B). As expected, and based on our previous observation^36^, we noted intense band above 250 kDa in *I. scapularis* (Is) whole tick lysate (Fig. 6B and Supplementary Fig. 6). These results show that anti-EL-6 antibody also recognizes *H. longicornis* OATP protein (KAH9381876.1). We then explored whether treatment with anti-EL6 antibody has any effect on *A. phagocytophilum* burden in *H. longicornis* ticks. We noticed significant reduction in the bacterial burden in *A. phagocytophilum*-infected *H. longicornis* ticks treated with EL-6 antibody when compared to control IgG-treated groups (Fig. 6C). In summary, these results indicate that treatment with anti-EL-6 antibody impairs *A. phagocytophilum* colonization in *H. longicornis* ticks.

## Discussion

Organic anion transporting polypeptides (OATPs) are a family of highly conserved transporters in arthropods like ticks, lice, and mosquitoes^41^. *Ixodes scapularis* tick express nine OATPs^41,43^. Our previous studies indicated that OATPs are critical for *A. phagocytophilum* survival and transmission from these ticks to the vertebrate host^34,36,37,39,40^. *Ixodes scapularis* and *I. pacificus* are the common vectors for *A. phagocytophilum*^17–20^. However, recent studies have indicated the presence of *A. phagocytophilum* in *H. longicornis* ticks collected from the field^8,9^. In this study, we noted that *H. longicornis* OATPs are also important for *A. phagocytophilum* survival in these ticks.

The role of *H. longicornis* molecules in the interactions with *A. phagocytophilum* is not known. As OATPs are highly conserved in different ticks and are critical for *A. phagocytophilum* survival in *I. scapularis* ticks, we reasoned whether these important molecules have any role in the interactions of *H. longicornis* ticks with this bacterium. Screening of *H. longicornis* genome with OATP signature sequence (WXGXWWXG) resulted in the identification of six OATPs. Along with the conserved tryptophan and glycine residues, we noted that all six *H. longicornis* OATPs have conserved alanine residues in the OATP signature sequence. The significance of this conserved alanine residue in the OATP signature sequence is currently not understood. In addition to the six OATPs analyzed in this study, we also noted that a hypothetical protein (GenBank Acc. No. KAH9381873.1) was annotated as OATP. However, OATP signature sequence was absent in this sequence. Therefore, we excluded KAH9381873.1 from the current analysis. High degree of percent identity of *H. longicornis* OATPs with other tick OATPs, including *I. scapularis*, suggests a conserved functional role for these important molecules in various ticks.

We noted variable levels of OATP transcripts at different developmental stages of *H. longicornis* ticks. The observation of significantly lower trend of all analyzed *H. longicornis* OATP transcripts in adult ticks compared to the levels noted in larvae or nymphal stages of these ticks suggest that these molecules are important in the immature stages of ticks. Larval and nymphal *H. longicornis* ticks acquire pathogens by feeding on infected animals. In addition, OATPs are important for blood feeding^36,44^. Therefore, it could be reasoned that increased levels of *H. longicornis* OATP expression in larval or nymphal ticks could facilitate blood feeding and/or acquisition and transmission of pathogens from and to the vertebrate host, respectively.

*Haemaphysalis longicornis* OATPs contain several posttranslational sites in their amino acid sequences including glycosylation sites. In our previous study, we noted that *I. scapularis* OATP (IsOATP4056) that has a predicted molecular mass of ~ 100 kDa was detected at above 250 kDa in an immunoblot performed with EL-6 and EL-2 antibody^36^. Treatment with deglycosylation mix did not impact the molecular mass of IsOATP4056 suggesting that this protein could exist in an oligomerized form or in other posttranslational-modified form^36^. *Haemaphysalis longicornis* OATP (GenBank Acc. No. KAH9381876) is an ortholog of IsOATP4056. The epitope region that was selected for the generation of EL-6 antibody is highly conserved in *H. longicornis* OATP (GenBank Acc. No. KAH9381876). Therefore, the observation of *H. longicornis* OATP (GenBank Acc. No. KAH9381876) at more than 250 kDa in an immunoblot suggests that this OATP may also be present in oligomerized and/or posttranslational-modified form. The observation of band above 250 kDa when SDS-PAGE analysis was performed with both 8% or 12% gel and in non-reducing and no-boiling conditions further supports that *H. longicornis* OATP (GenBank Acc. No. KAH9381876) may exist as an oligomerized form. In addition, the observation of multiple bands in both *H. longicornis* and *I. scapularis* total protein lysates (performed in both non-reducing and reducing conditions) is consistent with the observation of mammalian OATPs that forms dimers/homo or hetero-oligomers^45,46^. Furthermore, the presence of N-myristoylation sites, casein kinase II phospho sites, N-glycosylation sites, cAMP-and cGMP-dependent phospho sites or protein kinase C phospho sites indicate multiple kinds of posttranslational modifications alone or in combination could also contribute for the increased molecular mass of *H. longicornis* OATP (GenBank Acc. No. KAH9381876).

Our previous studies reported that there is a strong interplay between OATPs and tryptophan pathway^34–37,39–41^. We noted that exogenous addition of XA upregulated *isoatp4056* expression^40^. Knockdown of kynurenine amino transferase (KAT, enzyme that catalyzes formation of XA), via RNAi impacted *isoatp4056* expression^40^. We also reported that XA indirectly could facilitate transcriptional activation of *isoatp4056* gene at the promoter level^40^. These data prompted us to explore whether XA has any impact on *H. longicornis* OATP’s expression. Our results indicated differential regulation of *H. longicornis* OATPs. Interestingly, the expression of *H. longicornis* OATP (GenBank Acc. No. KAH9381876.1) was upregulated upon exogenous treatment with XA. These results indicate a conserved role for XA and OATP pathway in tick-*A. phagocytophilum* interactions. Some *H. longicornis* OATPs (GenBank Acc. Nos. KAH9381028.1 and KAH9381025.1) were noted to be downregulated in the presence of exogenous XA. However, *H. longicornis* OATPs (GenBank Acc. Nos. KAH9381028.1 and KAH9381025.1) transcript levels were not altered upon *A. phagocytophilum* infection when compared to the levels noted in the uninfected controls. The significance of the downregulation of these genes in the presence of exogenous XA is not known. Future studies on these aspects would provide more details on the role of XA in different physiological processes in these ticks.

We noted significantly decreased *A. phagocytophilum* loads in *I. scapularis* ticks fed on mice passively immunized with EL-6 antibodies^36^. In addition, we noted significant decrease in *A. phagocytophilum* loads in ISE6 tick cells upon treatment with EL-6 antibody^36^. The observation of reduced *A. phagocytophilum* burden in *H. longicornis* ticks upon treatment with EL-6 antibody further supports our previous observations with *I. scapularis* ticks and ISE6 tick cell line^36^. Moreover, these data support that IsOATP4056 and KAH9381876.1 that are orthologs in *I. scapularis* and *H. longicornis* ticks, respectively, aids in the survival/colonization of *A. phagocytophilum* in both these ticks. Our future studies are aimed in deciphering significance of OATP-tryptophan pathways in *H. longicornis*-pathogen interactions.

*Haemaphysalis longicornis* ticks are dramatically expanding their colonies in the United States. Studies like these are not only important to address some of the important vector biological questions but also could lead us in the development of strategies to target *H. longicornis* ticks and pathogens they transmit. In summary, our study suggests that treatment with EL-6 antibody could be an ideal strategy to target *A. phagocytophilum* colonization in different ticks.

## Methods

### Bacteria

*Anaplasma phagocytophilum* isolate NCH-1 (obtained from BEI Resources, NIAID, NIH), referred as *A. phagocytophilum,* was used throughout this study. *Anaplasma phagocytophilum* was maintained in HL-60 cells and isolated from these cells as described^37,42^. Briefly, infected HL-60 cells were centrifuged for 10 min at 2300 × *g* at 4 °C and the pellet was washed twice with sterile 1× phosphate buffer saline. Cells were then re-suspended in 4 ml 1× PBS and subjected to 8 quick pulses of sonication (8 s burst interspersed with 8 s rest periods using an ultrasonic sonicator on an amplitude setting of 30). Cells were then passed through 27-gauge needles for 6–8 times followed by freezing at − 80 °C for 15 min. After thawing, cells were centrifuged for 5 min at 700 × *g* at 4 °C and the supernatant was transferred to a fresh tube. The supernatant was centrifuged for 5 min at 1000 × g at 4 °C and the resulting supernatant was transferred to another fresh tube. This supernatant was then centrifuged for 10 min at 2300 × *g* at 4 °C. The pellet of host-cell free *A. phagocytophilum* (dense core, DC) was resuspended in 100 μl of 1× PBS and used for in vitro infection.

### Ticks

*Haemaphysalis longicornis* ticks (larvae, nymphs, adults) used in this study were obtained from BEI resources, CDC. Tick rearing was conducted in an Environmental Chamber from Parameter Generation and Control, USA. The incubator was set at 23 ± 2 °C with 94% relative humidity and 14:10 light:dark conditions. *Ixodes scapularis* (larvae) were obtained from BEI resources, CDC, and fed on uninfected C57BL/6J mice. Fed larvae were allowed to molt into nymphs. Total lysates from unfed nymphs were used in the immunoblotting assays.

### Antibody generation

IsOATP4056- EL-6 polyclonal antibodies used in this study were generated as described^36^ at a commercial facility (GenScript, USA). T*he I. scapularis* IsOATP4056 EL-6 epitope region was used for the generation of anti-EL6 antibody^36^.

### Total RNA, DNA isolation and qRT-PCR and qPCR data analysis

Total RNA from ticks (unfed/fed) was extracted using Aurum total RNA mini kit (BioRad, USA) following manufacturer instructions^37,47–49^. The cDNA was generated from total RNA using iScript cDNA synthesis kit (BioRad, USA)^37,47–49^, and used as template for the amplification of *H. longicornis oatps* and actin. DNA from ticks was extracted using DNeasy blood and tissue kit (Qiagen, USA). Oligonucleotides for actin were used from published study^50^. All other oligonucleotides used in this study are mentioned in Supplementary Table 2. qPCR and qRT-PCR was performed using iQ-SYBR Green supermix (BioRad, USA) or 2× Universal SYBR Green fast qPCR mix (ABclonal, USA) and CFX96 touch system (BioRad, USA)^37,47–49^. As an internal control and to normalize the amount of template, tick actin amplicons were quantified. The standard curves were prepared using tenfold serial dilutions starting from 1 to 0.000001 ng/μl of known quantities of respective gene fragments.

### Generation of *A. phagocytophilum*-infected *H. longicornis* nymphs

Unfed uninfected *H. longicornis* nymphs were bathed in 100 μl of *A. phagocytophilum*-DC preparation for 40 min at 34 °C. Ticks were washed three times with 1× PBS and stored in the tick environmental chamber for 1 week. After day 3, 5, 7 and 10 post infection, the nymphs were washed three times with 1× PBS, and the last wash of 1× PBS was saved as a negative control sample. Individual nymphs were processed for DNA extractions. DNA samples from the individual nymphs and the last 1× PBS wash sample were analyzed by qPCR to measure the bacterial loads.

### Tick experiments with xanthurenic acid (XA)

*Anaplasma phagocytophilum*-infected *H. longicornis* nymphs (day 7 post-infected) were used in this experiment. Stock of XA (10 mM) (Sigma, USA) was made in 0.5 N NaOH solution^34,37^. A 1:10 dilution of the stock was prepared in 1X PBS to a final concentration of 1 mM and used in all experiments. A mock solution was prepared in a similar way but without XA. *Anaplasma phagocytophilum*-infected *H. longicornis* nymphs were bathed in 500 μl of 100 μM XA or mock solution for 40 min at 34 °C. Ticks were washed 3 times with 1× PBS and stored in the tick environmental chamber for 24 h. Individual nymph was then processed further for DNA or RNA extractions followed by qPCR or qRT-PCR analysis to measure bacterial burden or *H. longicornis oatp* transcripts, respectively.

### Tick experiments with EL-6 antibody

*Anaplasma phagocytophilum*-infected *H. longicornis* nymphs were bathed in 500 μl of 1× PBS containing 5 μg of EL-6 antibody or control IgG antibody for 40 min at 34 °C. Ticks were washed 3 times with 1× PBS and stored in the tick environmental chamber for 24 h. Individual nymph was then processed further for DNA extractions followed by qRT-PCR analysis to measure the bacterial loads.

### Western blotting assay

Immunoblotting was performed as described^48^. Five *A. phagocytophilum*-infected unfed *H. longicornis* and five *A. phagocytophilum*-infected unfed *I. scapularis* nymphs were crushed and homogenized using pellet pestle cordless motor (Biospec, OK) and pellet pestle (VWR, USA) in modified-RIPA lysis buffer supplemented with EDTA-free protease inhibitor cocktail to generate tick lysates. Protein concentrations were determined by Bradford (BCA) protein assay kit (Pierce, USA) and as per the manufacturer’s recommendations. Tick lysates (10 μg) were mixed with Laemmli sample buffer, boiled for 5 min, and resolved on 8% or 12% SDS-PAGE gel (in reducing and boiling or non-reducing and no boiling conditions). Gel was run at 110 V and stained with Ponceau-S dye, destained with water, and then transferred to nitrocellulose membrane. Ponceau-S-stained image served as loading control. Membrane was blocked with 5% BSA in 1× TBST (1X TBS, 0.05% Tween 20). EL-6 antibody was used at a dilution of 1:500 in 5% BSA in 1× TBST. HRP-conjugated goat anti-rabbit secondary antibody was used at a dilution of 1:5000 in 5% BSA in 1× TBST. Development of the chemiluminescent substrate was visualized using a BioRad ChemiDoc Touch Imaging System (BioRad, USA).

### Sequence alignment and bioinformatic analysis

GenBank accession numbers for the sequences used in this study are mentioned in Supplementary Table 3. Amino acid sequence alignments for *H. longicornis* OATPs and orthologs from various organisms were performed using DNASTAR CLUSTALW alignment software. Sequence analyses were performed for *H. longicornis* OATPs with various organisms. The phylogenetic tree was constructed using the neighbor-joining method (BIONJ) using BIONJ algorithm in DNSTAR. The protein sequences for *H. longicornis* OATPs were downloaded from GenBank and individually analyzed at the PROSITE (http://prosite.expasy.org/) for the prediction of N-glycosylation, myristoylation, protein kinase C phosphorylation, casein kinase II phosphorylation and cAMP or c-GMP-dependent protein kinase phosphorylation sites and as described^37,51,52^. The number of post-translational modification sites in *H. longicornis* and *I. scapularis* are shown in Supplementary Table 4.

### Statistical analysis

Statistical significance in the data sets was analyzed using GraphPad Prism6 software (https://www.graphpad.com/) and Microsoft Excel 2010 (https://www.microsoft.com). A student’s *t*-test was used to compare statistical significance between the groups, or ANOVA was used to compare the variations in the groups. P values of < 0.05 were considered significant in all analyses.

### Ethics statement

*Ixodes scapularis* larvae feeding on mice was performed in accordance with University of Tennessee, Knoxville Institutional Animal Care and Use Committee (IACUC, animal assurance number: D16-00397) and approved protocol 2801-0221. The tick feeding experimental protocol was approved by the UTK, IACUC committee. The study was performed in accordance with ARRIVE guidelines. Acepromazine was used as a tranquilizer and was administered to animals prior to placement of larvae on mice and all efforts were made to minimize suffering.

### Supplementary Information

Supplementary Information.

## Data Availability

All data generated or analyzed during this study are included in this article and its supplementary information files.
