# Supplementary material for: Antibody-blocking of a tick transporter impairs Anaplasma phagocytophilum colonization in Haemaphysalis longicornis ticks
Source: Sci Rep. 2024 Apr 18;14:9003. doi: 10.1038/s41598-024-59315-w (PMC11026487; doi:10.1038/s41598-024-59315-w)
Supplement: Supplementary file 1 — Supplementary Information. [file 41598_2024_59315_MOESM1_ESM.pdf]

Antibody-blocking of a tick transporter impairs *Anaplasma phagocytophilum* colonization in *Haemaphysalis longicornis* ticks

Prachi Namjoshi [1], Donald M. Lubembe [2], Hameeda Sultana [1], and Girish Neelakanta [1, $]

Supplementary information

Supplementary figure legends

Supplementary Figure 1. Sequence distances and percent identity between *H. longicornis* OATPs and orthologs from other organisms. The percent identity (horizontally above black boxed diagonal line) and percent similarity (vertically below black boxed diagonal line) of the *H. longicornis* OATPs KAH9381028.1 (A), KAH9381027.1 (B) and KAH9381876.1 (C) sequence in comparison to the ortholog proteins from *Dermacentor andersoni* (Da), *Hyalomma asiaticum* (Ha), *Homo sapiens* (Hs)*, Ixodes scapularis* (*Is*), *Mus musculus* (Mm) and *Rhipicephalus sanguineus* (Rs) are shown. Sequence distance data were generated based on the CLUSTALW alignment of the sequences in DNASTAR. GenBank accession numbers for the sequences are mentioned along with the organism names.

Supplementary Figure 2. Sequence distances and percent identity between *H. longicornis* OATPs and orthologs from other organisms. The percent identity (horizontally above black boxed diagonal line) and percent similarity (vertically below black boxed diagonal line) of the *H. longicornis* OATPs KAH9365504.1 (A), KAH9380884.1 (B) and KAH9381025.1 (C) sequence

in comparison to the ortholog proteins from *Dermacentor andersoni* (Da), *Hyalomma asiaticum* (Ha), *Homo sapiens* (Hs)*, Ixodes scapularis* (*Is*), *Mus musculus* (Mm) and *Rhipicephalus sanguineus* (Rs) are shown. Sequence distance data were generated based on the CLUSTALW alignment of the sequences in DNASTAR. GenBank accession numbers for the sequences are mentioned along with the organism names.

Supplementary Figure 3. Phylogenetic analysis of *H. longicornis* OATPs. Phylogenetic tree of *H. longicornis* OATPs KAH9381028.1 (A), KAH9381027.1 (B) and KAH9381876.1 (C) with ortholog proteins from *Dermacentor andersoni* (Da), *Hyalomma asiaticum* (Ha), *Homo sapiens* (Hs)*, Ixodes scapularis* (*Is*), *Mus musculus* (Mm) and *Rhipicephalus sanguineus* (Rs) is shown. The phylogenetic tree was generated in DNASTAR using the Neighbor-Joining (BIONJ) method with BIONJ algorithm.

Supplementary Figure 4. Phylogenetic analysis of *H. longicornis* OATPs. Phylogenetic tree of *H. longicornis* OATPs KAH9365504.1 (A), KAH9380884.1 (B) and KAH9381025.1 (C) with ortholog proteins from *Dermacentor andersoni* (Da), *Hyalomma asiaticum* (Ha), *Homo sapiens* (Hs)*, Ixodes scapularis* (*Is*), *Mus musculus* (Mm) and *Rhipicephalus sanguineus* (Rs) is shown. The phylogenetic tree was generated in DNASTAR using the Neighbor-Joining (BIONJ) method with BIONJ algorithm.

Supplementary Figure 5. PCR amplification of *p44* to confirm *A. phagocytophilum* infection in *H. longicornis* nymphs bathed in *A. phagocytophilum*-DC (Ap-DC) preparation. A)

Agarose gel image showing PCR amplification product of the *p44* fragments in DNA isolated from individual *I. scapularis* or *H. longicornis* nymphs bathed in (Ap-DC) preparation is shown. The boxed band size of 334 bp is the expected size of *p44* gene fragment. The p44 standard (std) band was loaded as positive control. Final 1X PBS wash solution (collected after final wash of ticks) was used as a negative control. M indicates the DNA marker and NTC indicates no template control. B) qPCR analysis showing levels of *A. phagocytophilum* in *I. scapularis* (Is) and *H. longicornis* (Hl) ticks at day 7 post infection.

Supplementary Figure 6. Immunoblotting analysis of *H. longicornis* OATP. Immunoblotting analysis with EL-6 antibody and 8% SDS-PAGE showing levels of KAH9381876.1 or IsOATP4056 (indicated by black arrow) in uninfected unfed *H. longicornis* and *I. scapularis* tick lysates, respectively is shown. M indicates protein marker. Non reducing indicates non-reducing and no boiling conditions. *Hl* indicates *H. longicornis* and *Is* indicates *I. scapularis*. Ponceau S-stained gel image for total protein profile serves as a loading control in the immunoblotting analysis.

A

			% Identity						
		Accession Number	A	B	C	D	E	F	G
% Similarity	A	XP_050026642.1_Da		89.11	28.1	63.83	27.67	88.89	77.56
	B	KAH6928996.1_Ha	94.34		28.32	64.27	27.45	91.72	79.74
	C	NP_037404.2_Hs	43.36	44.01		27.67	93.46	29.63	27.67
	D	XP_029845044.2_Is	74.51	75.38	42.48		27.45	64.27	62.53
	E	NP_001033732.1_Mm	44.23	44.66	95.21	42.48		28.54	27.23
	F	XP_037519747.1_Rs	94.99	95.42	43.79	75.16	44.23		78.87
	G	KAH9381028.1_Hl	88.24	89.32	43.57	74.73	44.01	88.02	

B

			% Identity						
		Accession Number	A	B	C	D	E	F	G
% Similarity	A	XP_050026644.1_Da		86.53	25.93	67.68	24.58	88.55	79.12
	B	KAH6928998.1_Ha	89.9		25.08	65.66	22.9	89.39	77.95
	C	NP_001138516.1_Hs	41.58	42.09		26.26	35.02	25.42	26.09
	D	XP_029845043.2_Is	80.47	78.28	40.91		25.42	67	67.68
	E	NP_766429.2_Mm	43.1	41.92	54.21	44.44		23.57	24.75
	F	XP_037519748.1_Rs	92.76	91.41	42.59	80.64	42.93		79.12
	G	KAH9381027.1_Hl	86.87	84.51	42.26	80.81	44.61	87.04	

C

			% Identity						
		Accession Number	A	B	C	D	E	F	G
% Similarity	A	XP_050027613.1_Da		87.06	33.33	91.21	34.16	99.34	85.24
	B	KAH6928107.1_Ha	88.39		30.18	80.76	31.01	87.23	78.28
	C	KAI2575916.1_Hs	52.24	47.76		32.67	93.86	33.33	30.85
	D	QLF98518.1_Is	95.69	86.24	52.24		33.5	91.21	83.42
	E	NP_076397.2_Mm	53.23	48.59	95.19	53.23		34.16	31.51
	F	XP_037519415.1_Rs	99.34	88.56	52.24	95.85	53.07		85.24
	G	KAH9381876.1_Hl	88.56	81.92	49.25	88.06	50.08	88.56	

Supplementary Figure 1

A

			% Identity						
		Accession Number	A	B	C	D	E	F	G
% Similarity	A	XP_050035665.1_Da		84.06	40.63	64.06	40.31	82.5	70.63
	B	KAH6929534.1_Ha	92.19		40.63	65	40.63	88.13	72.81
	C	KAI2595578.1_Hs	57.5	58.13		39.38	80.94	40.63	41.88
	D	XP_040071371.1_Is	78.44	79.06	56.88		39.69	65.31	62.19
	E	NP_001342147.1_Mm	57.81	57.5	88.44	56.25		41.88	42.19
	F	XP_037518161.1_Rs	90.63	93.13	58.75	79.69	59.69		73.13
	G	KAH9365504.1_Hl	82.5	83.13	57.81	76.56	58.44	82.81	

B

			% Identity						
		Accession Number	A	B	C	D	E	F	G
% Similarity	A	XP_050026765.1_Da		88.05	26.02	70.54	27.17	90.83	76.43
	B	KAH6928437.1_Ha	92.64		25.04	68.41	26.19	91.33	75.45
	C	XP_005254946.1_Hs	44.19	42.88		28.31	34.86	26.19	27
	D	XP_029826927.3_Is	84.29	80.85	45.83		26.84	70.38	72.34
	E	NP_001239460.1_Mm	46.81	45.34	53.85	45.83		27.33	27.33
	F	XP_037520077.1_Rs	95.42	94.6	44.52	83.96	46.97		77.41
	G	KAH9380884.1_Hl	88.38	86.09	45.66	84.29	47.14	90.02	

C

			% Identity						
		Accession Number	A	B	C	D	E	F	G
% Similarity	A	XP_050026650.1_Da		92.52	23.99	79.44	23.99	95.17	88.01
	B	KAH6929001.1_Ha	96.42		24.3	78.5	24.3	95.02	87.23
	C	NP_059131.1_Hs	43.15	43.3		23.05	85.51	24.3	23.68
	D	XP_029845034.2_Is	87.07	86.76	40.81		23.21	79.75	81.62
	E	NP_067446.1_Mm	43.46	43.61	92.37	41.12		24.14	23.52
	F	XP_049272565.1_Rs	97.98	97.82	42.99	87.23	43.3		88.47
	G	KAH9381025.1_Hl	94.24	93.61	42.06	88.94	42.68	94.7	

Supplementary Figure 2

A

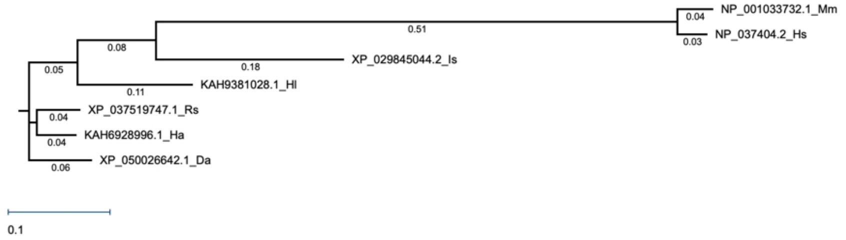

KAH9381028.1

B

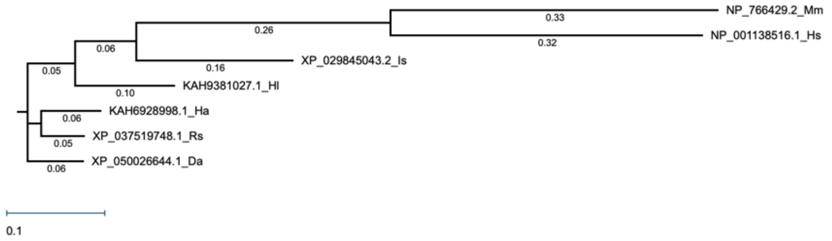

KAH9381027.1

C

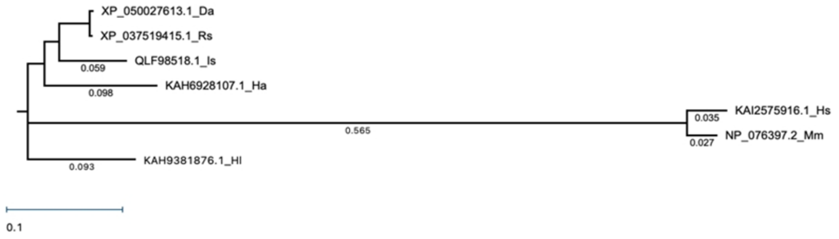

KAH9381876.1

Supplementary Figure 3

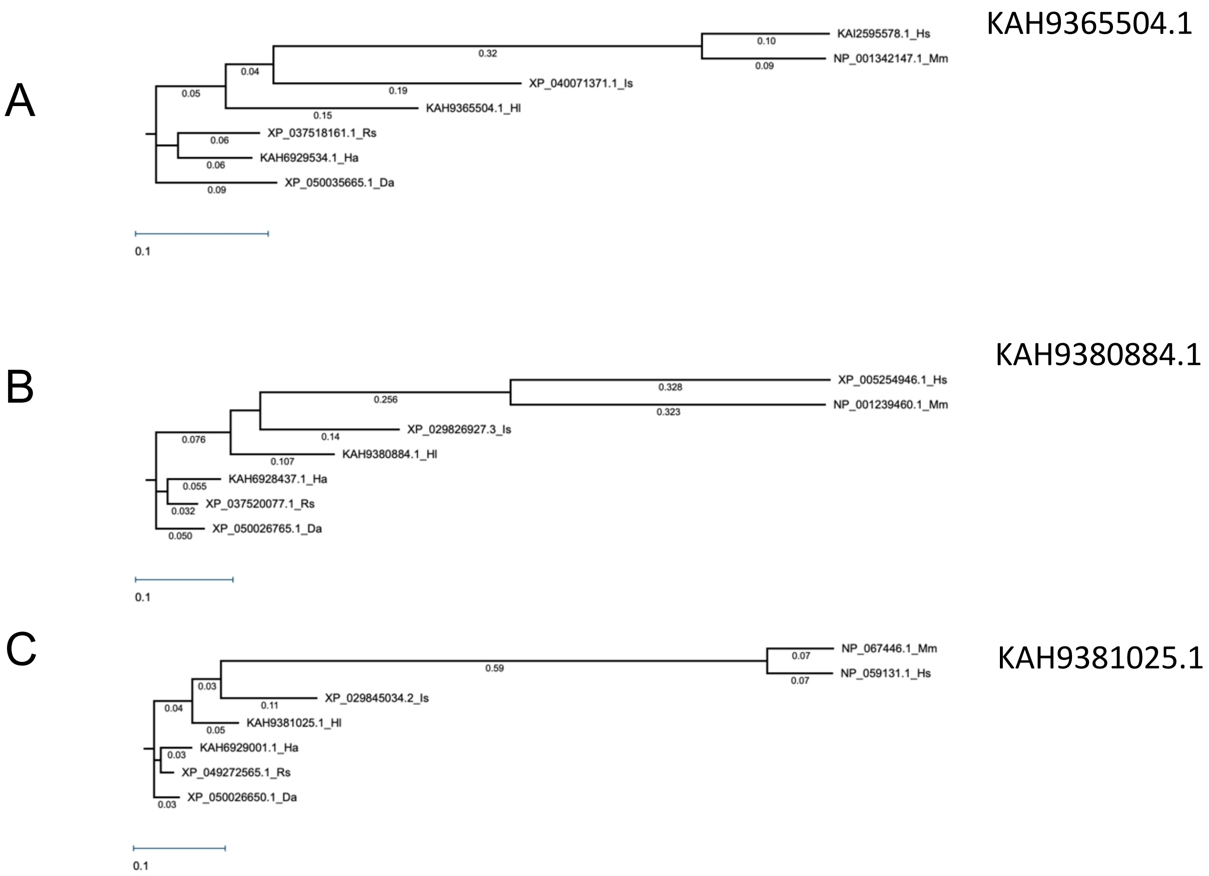

Supplementary Figure 4

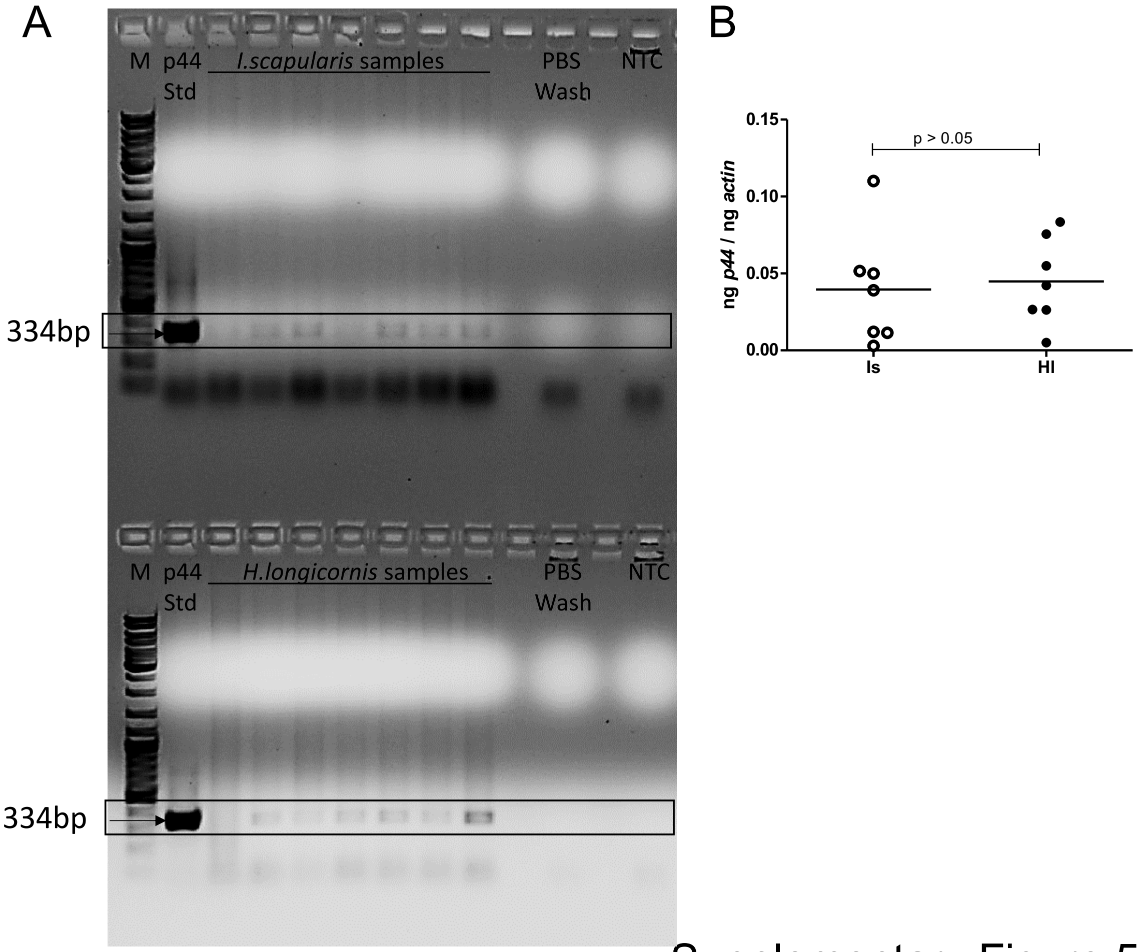

Supplementary Figure 5

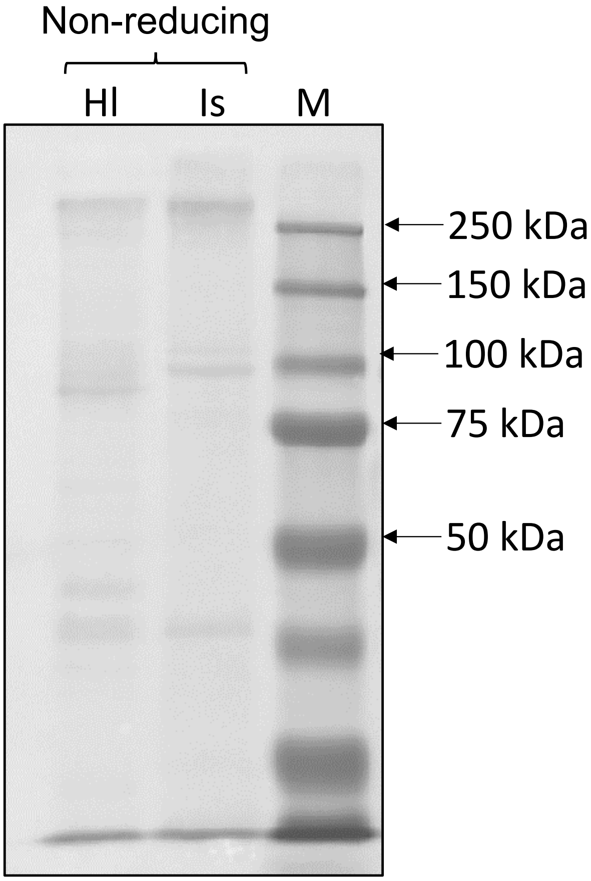

Ponceau S

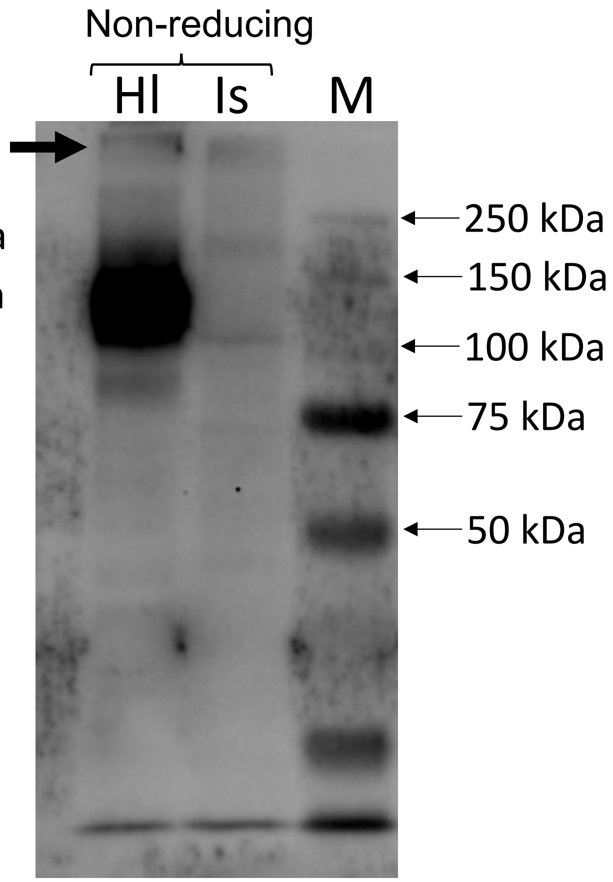

Immunoblot
Anti-EL6

Supplementary Figure 6

Supplementary Table 1. GenBank accession numbers for *H. longicornis* OATPs.

Accession no.	Annotation
KAH9381028.1	Hypothetical protein HPB48_008242 [*Haemaphysalis longicornis*]
KAH9381027.1	Hypothetical protein HPB48_008241 [*Haemaphysalis longicornis*]
KAH9381876.1	Hypothetical protein HPB48_015404 [*Haemaphysalis longicornis*]
KAH9365504.1	Hypothetical protein HPB48_016282 [*Haemaphysalis longicornis*]
KAH9380884.1	Hypothetical protein HPB48_012506 [*Haemaphysalis longicornis*]
KAH9381025.1	Hypothetical protein HPB48_008239 [*Haemaphysalis longicornis*]

Supplementary Table 2: Oligonucleotides used in this study.

Oligonucleotides (5’-3’)	Gene product
GATCCATCACGACCTACA	KAH9381028.1, Hypothetical protein HPB48_008242 [*Haemaphysalis longicornis*]
GTTTGGATTCCCAGAACTC	KAH9381028.1, Hypothetical protein HPB48_008242 [*Haemaphysalis longicornis*]
CATCTTCGACGCTACCT	KAH9381027.1, Hypothetical protein HPB48_008241 [*Haemaphysalis longicornis*]
GTCCACTTCTCCGTACAT	KAH9381027.1, Hypothetical protein HPB48_008241 [*Haemaphysalis longicornis*]
AACGGCATCACCTACTT	KAH9381876.1, Hypothetical protein HPB48_015404 [*Haemaphysalis longicornis*]
GAAGAGGAGCACCATGA	KAH9381876.1, Hypothetical protein HPB48_015404 [*Haemaphysalis longicornis*]
CTACCGCTACCTGTTCAT	KAH9365504.1, Hypothetical protein HPB48_016282 [*Haemaphysalis longicornis*]
GACGTCGGTGTAGATAGAG	KAH9365504.1, Hypothetical protein HPB48_016282 [*Haemaphysalis longicornis*]
GACAACATCCGCAAGAAG	KAH9380884.1, Hypothetical protein HPB48_012506 [*Haemaphysalis longicornis*]
CAGTATGTAGCCCATCCA	KAH9380884.1, Hypothetical protein HPB48_012506 [*Haemaphysalis longicornis*]
AGAGGGACATACGACAAC	KAH9381025.1, Hypothetical protein HPB48_008239 [*Haemaphysalis longicornis*]
CCTTGTCACGCTCATCTA	KAH9381025.1, Hypothetical protein HPB48_008239 [*Haemaphysalis longicornis*]

Supplementary Table 3: GenBank accession numbers for all the sequences analyzed in this study.

Protein	Accession number	Organism
Hypothetical protein HPB48_008242	KAH9381028.1	*Haemaphysalis longicornis*
	XP_029845044	*Ixodes scapularis*
	NP_037404	*Homo sapiens*
	NP_001033732	*Mus musculus*
	XP_037519747	*Rhipicephalus sanguineus*
	XP_050026642	*Dermacentor andersoni*
	KAH6928996	*Hyalomma asiaticum*
Hypothetical protein HPB48_008241	KAH9381027.1	*Haemaphysalis longicornis*
	XP_029845043	*Ixodes scapularis*
	NP_001138516	*Homo sapiens*
	NP_766429	*Mus musculus*
	XP_037519748	*Rhipicephalus sanguineus*
	XP_050026644	*Dermacentor andersoni*
	KAH6928998	*Hyalomma asiaticum*
Hypothetical protein HPB48_015404	KAH9381876.1	*Haemaphysalis longicornis*
	QLF98518.1	*Ixodes scapularis*
	KAI2575916	*Homo sapiens*
	NP_076397	*Mus musculus*
	XP_037519415	*Rhipicephalus sanguineus*
	XP_050027613	*Dermacentor andersoni*
	KAH6928107	*Hyalomma asiaticum*
Hypothetical protein HPB48_016282	KAH9365504.1	*Haemaphysalis longicornis*
	XP_040071371	*Ixodes scapularis*

	KAI2595578	*Homo sapiens*
	NP_001342147	*Mus musculus*
	XP_037518161	*Rhipicephalus sanguineus*
	XP_050035665	*Dermacentor andersoni*
	KAH6929534	*Hyalomma asiaticum*
Hypothetical protein HPB48_012506	KAH9380884.1	*Haemaphysalis longicornis*
	XP_029826927	*Ixodes scapularis*
	XP_005254946	*Homo sapiens*
	NP_001239460	*Mus musculus*
	XP_037520077	*Rhipicephalus sanguineus*
	XP_050026765	*Dermacentor andersoni*
	KAH6928437	*Hyalomma asiaticum*
Hypothetical protein HPB48_008239	KAH9381025.1	*Haemaphysalis longicornis*
	XP_029845034	*Ixodes scapularis*
	NP_059131	*Homo sapiens*
	NP_067446	*Mus musculus*
	XP_049272565	*Rhipicephalus sanguineus*
	XP_050026650	*Dermacentor andersoni*
	KAH6929001	*Hyalomma asiaticum*

Supplementary Table 4: Number of posttranslational modifications in OATP orthologs in *H. longicornis* and *I. scapularis*.

Organism	Accession	cAMP- and cGMP-dependent phospho	Protein kinase C phosphor sites	N-glycosylation	Casein kinase II phospho	N-myristoylation
Haemaphysalis longicornis	KAH9381028.1	3	9	5	11	19
	KAH9381027.1	5	6	4	12	20
	KAH9381876.1	1	4	8	15	17
	KAH9365504.1	0	6	4	8	12
	KAH9380884.1	2	8	3	6	16
	KAH9381025.1	3	14	4	8	14
Ixodes scapularis	XP_029845044	4	4	2	5	15
	XP_029845043	2	9	6	11	21
	QLF98518.1	1	4	6	16	15
	XP_040071371	0	4	0	2	4
	XP_029826927	1	6	4	8	16
	XP_029845034	4	15	5	8	11
